# Chrononutritional regulation of metabolic homeostasis: intermittent fasting through gut microbiota, circadian rhythms, and autophagy

**DOI:** 10.3389/fnut.2026.1964828

**Published:** 2026-10-02

**Authors:** Mengye Ma, Jiami Yuan, Feng Xu, Ying Zhou, Tao Li, Dezheng Zhou, Xiaohong Zhang

**Affiliations:** 1 School of Public Health, Health Science Center, Ningbo University, Zhejiang Key Laboratory of Pathophysiology, Ningbo, Zhejiang, China; 2 Department of Gastroenterology, the Affiliated Lihuili Hospital of Ningbo University, Ningbo, Zhejiang, China; 3 HEALTH BioMed Research & Development Center, Health BioMed Co. Ltd., Ningbo, Zhejiang, China

**Keywords:** autophagy, circadian rhythm, glucose and lipid metabolism disorder, gut microbiota, intermittent fasting, obesity

## Abstract

The global obesity epidemic has increased the burden of metabolic diseases. Dietary interventions are key to managing obesity and improving metabolic health. Intermittent fasting (IF) has emerged as a promising chrononutritional intervention that may modulate metabolic metabolic homeostasis through timed eating–fasting cycles. This review examines four main IF patterns:time-restricted feeding (TRF), alternate day fasting (ADF), the 5:2 fasting, and the 4:3 fasting, and evaluates their effects on body weight, glucose homeostasis, insulin sensitivity, and lipid profiles. Furthermore, it delineates the mechanistic pathways through which IF confers metabolic benefits beyond caloric restriction alone: (1) alleviating insulin resistance by enhancing the intestinal mucosal barrier and upregulating GLP-1 expression suppressing endotoxemia-related inflammation; (2) restoring circadian rhythmicity in the expression of metabolism-related genes to correct metabolic dysregulation; (3) increasing energy expenditure and lipid oxidation via short-chain fatty acid-mediated browning of white adipose tissue (WAT); and (4) enhancing cellular autophagy through activation of the SIRT3–AMPK–mTOR signaling pathway, thereby improving overall metabolic function. Evidence supports IF as a potentially effective alternative for obesity and metabolic disorders through chronobiological mechanisms. However, potential adverse effects like hypoglycemia and adherence challenges highlight the need for individualized approaches in clinical practice.

## 1 Introduction

The rising prevalence of obesity and associated metabolic disorders has intensified interest in dietary strategies that target not only energy balance but also the timing of food intake. According to the World Health Organization (WHO), 39% of the global adult population was overweight in 2020, with over 600 million individuals classified as clinically obese, a figure that has nearly doubled since 2000 (1). Projections suggest that global obesity prevalence may exceed 50% by 2030, driving substantial increases in diabetes and cardiovascular diseases, with estimated annual additional healthcare costs surpassing US$1.2 trillion (1). In this context, chrononutrition, an emerging strategy that prioritizes the timing of food intake relative to circadian rhythms, has gained recognition as a promising approach to metabolic health. Intermittent fasting (IF), characterized by cyclical periods of eating and fasting, represents the most widely studied dietary paradigm within this chronobiological framework. Calorie restriction (CR) has well-established efficacy in weight management and metabolic regulation (2), but its long-term effectiveness is limited by poor adherence, which undermines its clinical impact (3). From a chrononutrition perspective, IF represents a paradigm shift: rather than merely restricting total calories, it imposes temporal patterns of feeding and fasting that may synchronize metabolic rhythms with endogenous circadian clocks (4). The concept of IF dates back to 1915, when Folin proposed short-term fasting as a safe method for weight reduction (5). In 1946, Carlson first introduced the concept of IF in rodent lifespan studies (6), although its underlying mechanisms were not comprehensively investigated until the early 21st century, following advances in molecular biology techniques. While IF and CR achieve comparable weight loss in many trials, IF may confer additional metabolic benefits through chronobiological mechanisms that operate independently of caloric restriction (2, 7, 8). This review focuses on four widely adopted IF patterns: time-restricted feeding (TRF), alternate-day fasting (ADF), 5:2 fasting, and 4:3 fasting. We systematically review their effects on obesity and dysregulated glucose and lipid metabolism, and elucidate the underlying mechanisms including modulation of the gut microbiota, circadian rhythm reprogramming, browning of white adipose tissue (WAT), and activation of autophagy. Unlike prior reviews focused predominantly on energy deficit comparisons between IF and CR, this review uniquely synthesizes these chronobiological mechanisms into an integrated framework to explain the metabolic benefits of IF beyond caloric restriction, and identifies critical translational gaps limiting clinical application. Future research should prioritize large-scale randomized controlled trials to validate preclinical findings in diverse populations. Study designs that systematically isolate the chronobiological effects of IF from its energy-restrictive component are needed to clarify mechanism-specific benefits. Additionally, identification of predictive biomarkers including circadian phase markers, baseline gut microbiota composition, and autophagy-related metabolites will be essential for developing personalized IF protocols. Furthermore, investigations into synergistic effects between IF and complementary interventions such as physical exercise and pharmacotherapy may optimize therapeutic outcomes for metabolic disorders.

## 2 Method

To address these questions and provide a comprehensive overview, this review systematically synthesizes existing evidence on IF’s effects and underlying mechanisms. A systematic literature search was performed in PubMed and Web of Science from database inception to July 31, 2026, using the following search terms and their Boolean combinations: “intermittent fasting,” “time-restricted feeding,” “time-restricted eating,” “alternate-day fasting,” “5:2 fasting,” “4:3 fasting,” “continuous energy restriction,” “caloric restriction,” “glycemic control,” “insulin sensitivity,” “lipid metabolism,” and “circadian rhythm.” For human studies, inclusion criteria were: (1) randomized controlled trials (RCTs) or prospective cohort studies; (2) adult participants (≥18 years); (3) intervention duration of ≥2 weeks for acute metabolic endpoints or ≥4 weeks for weight-related outcomes; (4) evaluation of at least one outcome of interest (body weight, fasting glucose, HbA1c, insulin, or circulating lipids); and (5) articles published in English. Exclusion criteria comprised: (1) studies involving participants with concurrent malignancies, end-stage organ failure, or pregnancy; (2) observational studies without a comparator arm; and (3) reviews, case reports, conference abstracts, or editorials. For preclinical mechanistic evidence, we included studies that directly investigated the molecular pathways (intestinal barrier, circadian clock, adipose thermogenesis, or autophagy) in response to IF in rodent models, with priority given to those that reported both mechanistic readouts and corresponding metabolic endpoints. After title/abstract screening and full-text review, 76 eligible references were included in this synthesis (Figure 1).

**Figure 1:**
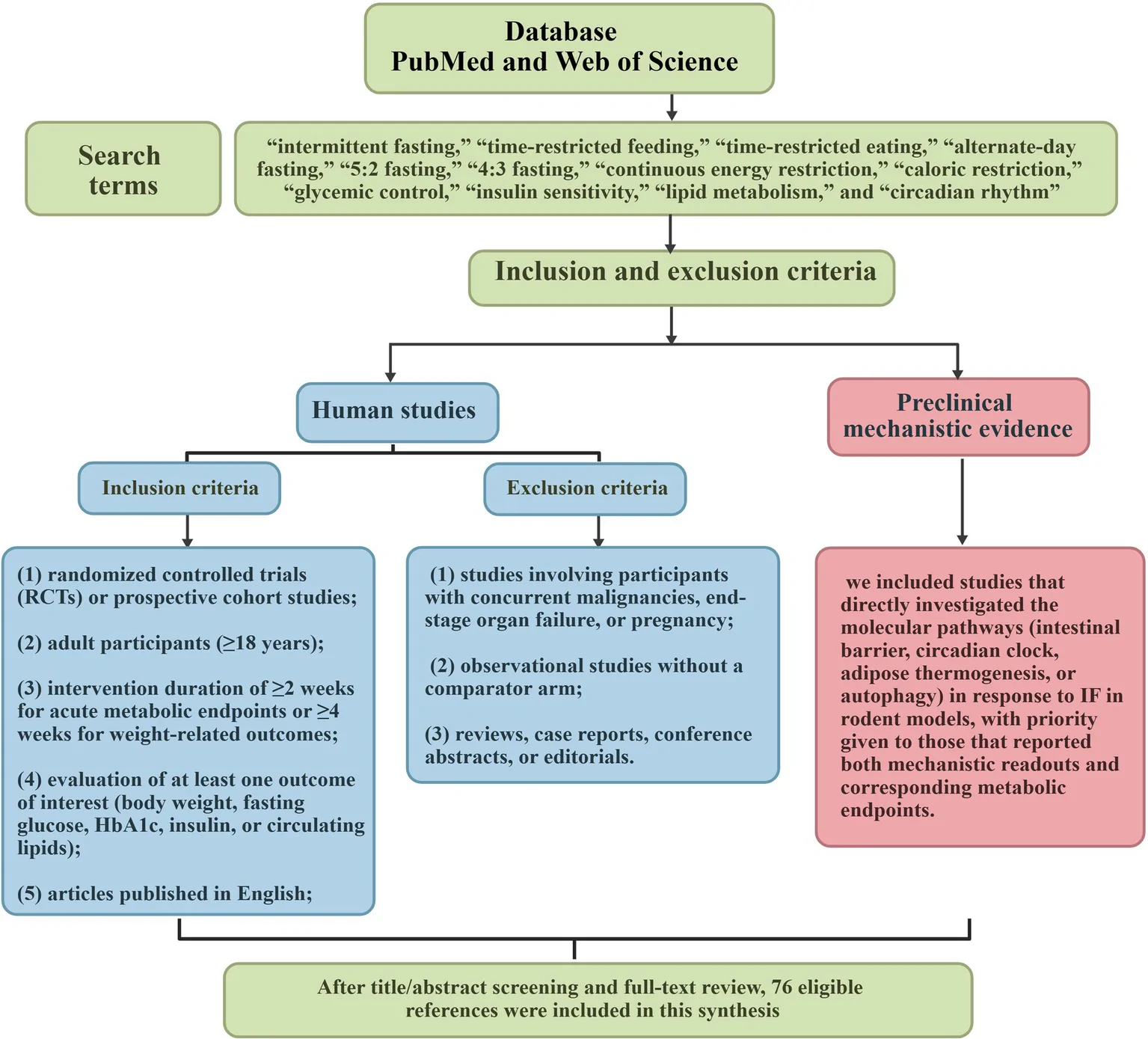
Inclusion and exclusion criteria. A systematic search in PubMed and Web of Science was performed using predefined terms. After applying inclusion/exclusion criteria for human and preclinical studies, 76 eligible references were included in the final synthesis.

## 3 Classification of intermittent fasting dietary patterns

Based on fasting duration and frequency, IF encompasses five main patterns defined by fasting duration and frequency: TRF, ADF 5:2 fasting, 4:3 fasting, and religious fasting (9, 10). TRF involves maintaining normal daily caloric intake within a defined eating window of 4–12 h each day, followed by fasting for the remainder of the day (11). Based on the eating window length, TRF is further classified into five patterns: 20:4, 18:6, 16:8, 14:10, and 12:12 regimens, with the 16:8 regimen being the most widely adopted TRF approach (4, 12). ADF involves alternating fasting days with ad libitum feeding days. On fasting days, energy intake is typically restricted to approximately 500 kcal or 20–25% of basal energy requirements, while feeding days have no time restrictions on food intake (10). The 4:3 fasting pattern consists of three low-calorie days per week (typically involving energy intake of around 20% of basal metabolic rate), with ad libitum eating on the other four days (13). Religious fasting refers to structured periods of abstinence observed in Christian, Islamic, and Buddhist traditions. Among these, TRF, ADF, 5:2, and 4:3 have been most extensively studied in obesity and glucose–lipid metabolic disorders, and thus constitute the focus of this review (Figure 2).

**Figure 2:**
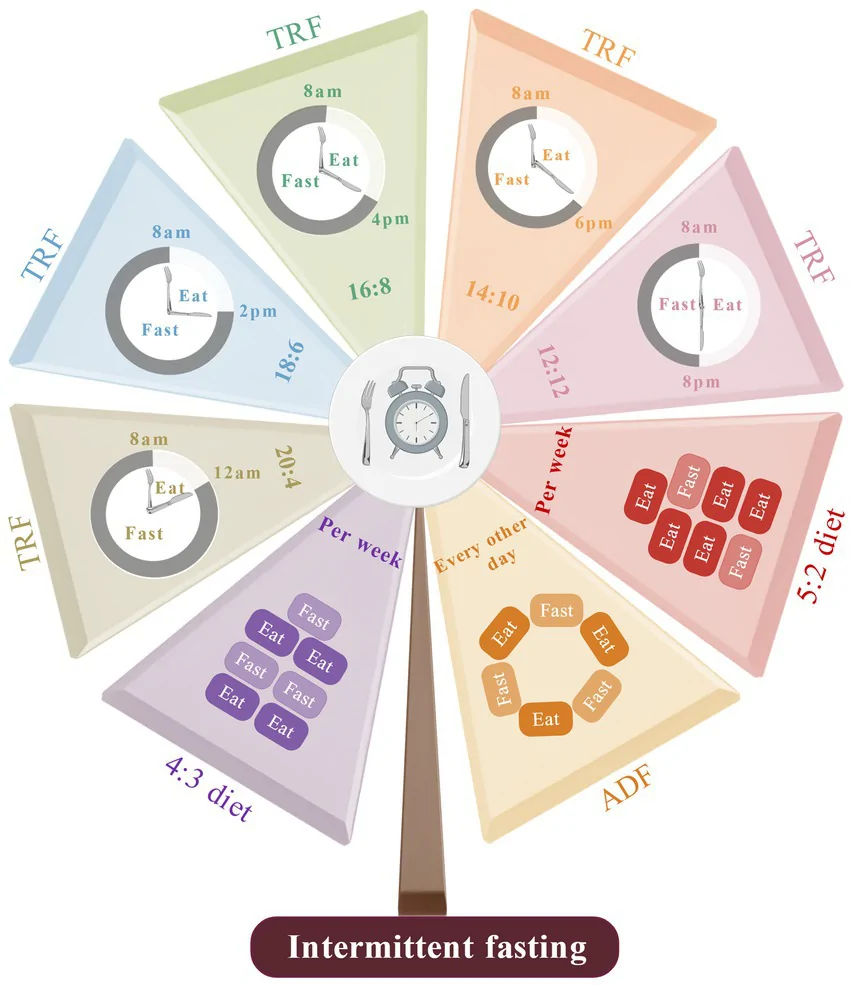
Common types of intermittent fasting (IF). Schematic overview of four IF patterns: TRF (16:8, 14:10, 20:4), ADF (alternating ad libitum feeding and fasting days), 5:2 (two low-calorie days per week), and 4:3 (three low-calorie days per week).

## 4 The weight loss effects of intermittent fasting dietary patterns

While IF has been extensively studied for weight management, this section briefly summarizes the key findings on body weight changes across different IF patterns, with a focus on their clinical relevance and limitations.

A recent network meta-analysis of 99 randomized trials concluded that most IF regimens achieve weight loss comparable to continuous energy restriction (CER) over the long term (≥24 weeks), despite short-term advantages for certain patterns such as ADF (14). Among the four main IF patterns, namely TRF, ADF, 5:2, and 4:3 fasting, typical weight reductions range from approximately 1.5 kg (TRF) to 7.7 kg (4:3), depending on intervention duration and adherence (13, 14).

Several factors influence the magnitude of weight loss. Eating window duration appears critical for TRF, with 6–8 h windows yielding greater reductions than 10–12 h windows. Adherence varies substantially across regimens: while TRF shows compliance rates of 85–88% at 12 weeks, long-term adherence to 5:2 fasting drops below 30% over two years (15). Combined interventions—particularly TRF with exercise or energy restriction—may enhance efficacy (16–18), though head-to-head comparisons with CER generally show no significant differences in weight outcomes (14, 19).

Collectively, IF represents an effective alternative to CER for weight management, with comparable long-term efficacy. However, the potential of IF to improve metabolic health may not reside in superior weight reduction, but rather in its chronobiological effects on glucose and lipid homeostasis, mechanisms that operate, at least in part, independently of caloric restriction and body weight changes, as elaborated in the following sections (14).

## 5 The regulatory effects of intermittent fasting on glucose and lipid metabolism

While the weight-loss effects of IF have been extensively reviewed elsewhere, a more clinically relevant question is whether IF may offer metabolic benefits that extend beyond, or are distinct from, those attributable to caloric restriction and weight loss alone. This section critically evaluates the human evidence for IF-mediated improvements in glucose and lipid homeostasis, with particular emphasis on studies that directly compare IF with CER, which is the gold standard for isolating fasting-specific from energy deficit-driven effects.

### 5.1 Glycemic benefits of IF: are they independent of caloric restriction, or chronobiologically driven?

Evidence from multiple randomized controlled trials indicates that various IF regimens, including TRF, ADF, and the 5:2 fasting, consistently reduce fasting glucose, fasting insulin, and HbA1c in overweight, obese, and diabetic populations (20–26). In patients with type 2 diabetes, for instance, the 5:2 fasting has been reported to achieve HbA1c reductions comparable to those of metformin or empagliflozin over 16 weeks (26), and early TRF (eating window restricted to before 15:00) has shown promise in improving insulin sensitivity in prediabetic individuals (8, 21).

However, when IF is directly compared with CER under isocaloric conditions, the picture becomes less clear. Two independent trials by Carter et al. demonstrated that 5:2 fasting and CER produced nearly identical HbA1c reductions over both 12 weeks and 12 months in patients with diabetes (27, 28). Similarly, in patients with NAFLD, 16:8 TRF and CER yielded comparable improvements in glycemic parameters at 6 and 12 months (29). These findings raise the possibility that the glycemic benefits of IF may be largely driven by overall energy deficit rather than by fasting-specific or chronobiological effects, at least in the populations studied to date.

Notably, the comparative effectiveness of IF versus CER may depend on the specific regimen and outcome measured. While ADF and 16:8 TRF appear equally effective in reducing fasting glucose in prediabetic individuals (4), ADF does not seem to influence glucose levels in healthy, non-obese adults, suggesting that baseline metabolic status modulates the response to IF (30, 31). Whether these differences reflect true chronobiological effects or simply variation in achieved energy deficit remains an open question that awaits mechanistic investigation.

### 5.2 Lipid metabolism: effects of IF and comparisons with CER

The effects of IF on circulating lipids are more variable than its glycemic effects. Some trials have reported modest reductions in total cholesterol, LDL-cholesterol, or triglycerides with TRF or 5:2 fasting (32, 33), while others have found no significant changes (34, 35). In patients with NAFLD, both 16:8 TRF and CER produced comparable reductions in intrahepatic triglyceride content at 6 months (−8.3% vs. − 8.1%) and 12 months (−6.9% vs. − 7.9%), with no significant differences between the two approaches (29).

Hepatic fat reduction has been more consistently observed with 5:2 fasting, with studies reporting decreases of 14.3 to 20.5% over 12 weeks, although direct comparisons with CER are lacking in these studies (36–39). Interestingly, one short-term TRF intervention (4 days) paradoxically increased LDL-cholesterol despite elevating HDL-cholesterol, highlighting the need for longer-term safety data (22, 40).

Collectively, the evidence for IF-specific effects on lipid metabolism remains inconclusive, with few studies demonstrating consistent advantages over CER under isocaloric conditions. If IF confers distinct lipid-modulating benefits, these are likely subtle and may require longer intervention periods or specific populations to emerge, and they may be mediated by mechanisms that are not captured by standard lipid panels (41).

In summary, the human evidence supports the efficacy of IF in improving glucose and lipid metabolism, with glycemic improvements being more consistently documented than lipid modulation. Among the four IF patterns reviewed, the 4:3 fasting has received the least attention, with only one 12-month trial reporting improvements in eating behaviors and elevations in adiponectin levels compared with daily CR (42); the absence of direct glucose or lipid measurements in this study precludes definitive conclusions about its metabolic efficacy.

More broadly, several lines of evidence suggest that the metabolic improvements observed with IF may be largely attributable to energy deficit, rather than to fasting-specific chronobiological effects: (1) head-to-head comparisons with CER generally show comparable metabolic outcomes (27–29); (2) the response to IF varies substantially across populations, with healthy individuals showing little or no glycemic benefit (30); and (3) direct evidence linking the fasting-specific mechanisms identified in preclinical studies, including circadian reprogramming, gut microbiota remodeling, adipose browning, and autophagy, to the metabolic improvements observed in humans remains limited. These observations do not diminish the potential clinical value of IF, which may offer advantages in terms of dietary adherence and individual preference, but they do sharpen the scientific question: if IF and CER achieve similar metabolic improvements despite different temporal eating patterns, what are the true chronobiological mechanisms that distinguish IF, and under what conditions do they become clinically relevant? This question forms the bridge to the following section, which examines the preclinical evidence for fasting-specific mechanisms.

## 6 Mechanisms by which intermittent fasting dietary patterns improve glucose and lipid metabolism

### 6.1 Insulin resistance improvement via intestinal barrier, inflammation suppression and GLP-1 secretion

Dysbiosis of the gut microbiota can cause metabolic disorders in the host and induce obesity and related diseases (43). Lipopolysaccharide (LPS), a component of the cell wall of Gram-negative bacteria, activates the TLR4 pathway, promoting the release of pro-inflammatory factors such as TNF-α and IL-6, inducing chronic low-grade inflammation, inhibiting insulin signaling, and leading to insulin resistance and hyperglycemia (44, 45). When the body is exposed to stress such as ischemia or infection, the intestinal mucosal barrier is damaged, permeability increases, and LPS enters the bloodstream, exacerbating systemic inflammation and disrupting glucose and lipid metabolism. Brun et al. found that in obese mice (ob/ob, db/db), the expression of intestinal tight junction proteins Occludin and ZO-1 was downregulated, and portal vein LPS significantly increased, forming “portal endotoxemia” (46). Cani et al. confirmed that a high-fat diet led to intestinal barrier damage in mice, a sharp increase in portal vein LPS, accompanied by microbiota dysbiosis (increase in Gram-negative/Gram-positive bacteria ratio) and an increase in total LPS in the intestinal lumen. LPS binds to TLR4 on adipose tissue macrophages, releasing inflammatory mediators and enhancing oxidative stress, inhibiting insulin signaling, leading to adipocyte hypertrophy and a 70% increase in body fat. After intervention with broad-spectrum antibiotics, microbiota diversity sharply decreased, beneficial bacteria such as *Lactobacillus* sharply declined, but intestinal permeability, plasma LPS and inflammatory indicators all recovered, and insulin sensitivity and weight gain were reversed (47). This indicates that a high-fat diet triggers a “microbiota-barrier-LPS” axis that initiates a “inflammation-oxidative stress-insulin resistance” cascade reaction, and targeting the microbiota or blocking LPS signaling can break this pathological cycle. Accumulating evidence indicates that IF improves insulin sensitivity, at least in part, by modulating this gut microbiota–intestinal barrier–endotoxemia axis (48). Guo et al. showed that patients with metabolic syndrome who followed a 5:2 fasting regimen for 8 weeks experienced an increase in plasma short-chain fatty acids (SCFAs) and a decrease in LPS, suggesting that IF enhances intestinal barrier function (48). Preclinical studies in rodent models have consistently demonstrated that IF enhances intestinal barrier function by upregulating tight junction proteins (Occludin, ZO-1, Claudin-1) and mucin MUC-2, which in turn reduces serum LPS levels and attenuates systemic inflammation (49–51). These effects are likely mediated by enrichment of beneficial bacteria (*Lactobacillus*, *Bifidobacterium*, *Akkermansia*) and SCFA production, which promote intestinal epithelial repair and barrier integrity (52). In addition to enhancing barrier function, SCFAs may stimulate GLP-1 secretion from enteroendocrine L-cells via *GPR41/43* activation, which directly improves insulin sensitivity and glycemic control (Figure 3).

**Figure 3:**
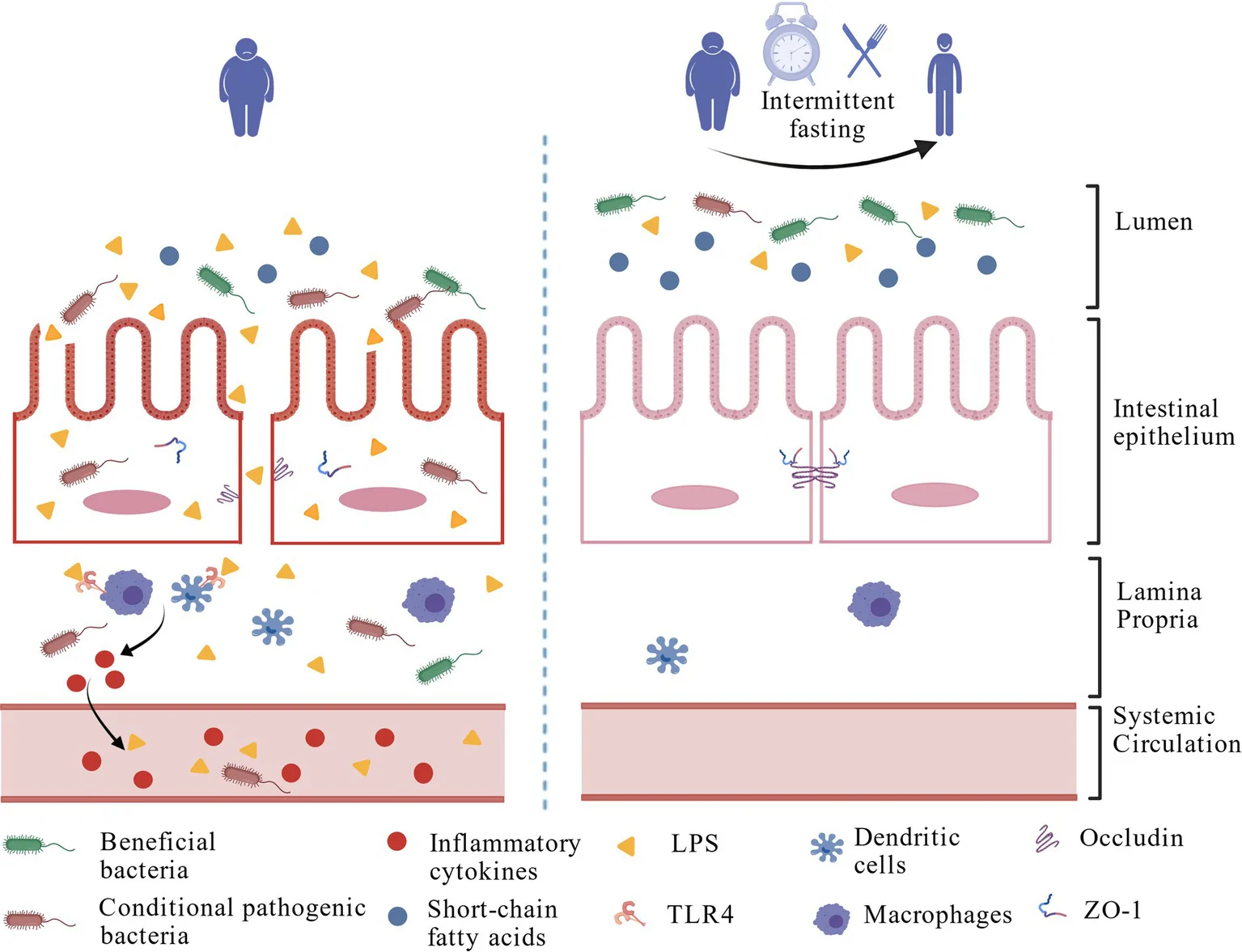
IF mitigates endotoxemia and systemic inflammation through modulation of the gut microbiota–intestinal barrier axis. IF modulates the gut microbiota–intestinal barrier–endotoxemia axis to improve glucose and lipid metabolism. IF enriches beneficial bacteria (*Lactobacillus, Bifidobacterium*, and *Akkermansia*) and SCFAs, while reducing conditional pathogenic bacteria. SCFAs upregulate tight junction proteins (Occludin, ZO-1) to preserve intestinal epithelial integrity, thereby limiting LPS translocation from the lumen into the lamina propria and systemic circulation. Reduced LPS levels suppress TLR4-mediated activation of macrophages and dendritic cells, decreasing the release of pro-inflammatory cytokines (TNF-α, IL-6) and ultimately alleviating insulin resistance and metabolic dysfunction.

Despite this supportive evidence, several critical limitations should be acknowledged. First, the majority of mechanistic details derive from preclinical rodent models, and direct evidence linking IF-induced microbiota changes to improved intestinal barrier function and insulin sensitivity in humans remains limited (53). Second, it is unclear whether the barrier-enhancing and GLP-1-stimulating effects of IF are driven by fasting-specific signals (circadian alignment, temporal patterns of SCFA production) or simply reflect reduced caloric intake, as no head-to-head mechanistic studies have compared IF with CER (54). Third, the relative contribution of GLP-1 versus barrier function to IF-mediated insulin sensitization in humans has not been systematically evaluated. Addressing these gaps will be essential to determine whether the gut microbiota–intestinal barrier axis represents a mechanism that is specifically engaged by IF, as opposed to a general consequence of energy restriction (54).

### 6.2 Circadian reprogramming of biological clock genes and gut microbiota rhythms

Circadian rhythms represent the intrinsic 24-h periodic patterns of physiological and behavioural changes within organisms, widely observed across animals, plants, and even microorganisms. These rhythms influence diverse vital functions, including sleep, body temperature, hormone secretion, and metabolism (54). This rhythm is regulated by the suprachiasmatic nucleus (SCN) in the hypothalamus. The SCN receives light signals from the retina, activating the expression of clock genes such as *CLOCK* and *BMAL1*. The proteins encoded by these genes form complexes that activate the transcription of *PER* and *CRY* genes. Subsequently, *PER* and *CRY* proteins inhibit the activity of the CLOCK-BMAL1 complex, forming a negative feedback loop that maintains the 24-h rhythm (55). Unhealthy dietary patterns and caloric intake disrupt circadian rhythms, leading to adverse health outcomes such as obesity and type 2 diabetes (56).

IF improves and restores metabolic rhythms by modulating the expression of circadian clock genes, thereby inhibiting metabolic disorders (57, 58). In the study by Damiola et al., preclinical studies have demonstrated that TRF resets circadian gene expression in peripheral tissues. Damiola et al. showed that restricting food access to the daytime (inactive phase) in mice nearly inverted the expression phases of hepatic clock genes (*Per1*, *Per2*, *Per3*, *Cry1*, *Rev-erbα*) and synchronized peripheral oscillators across the kidney, heart, and pancreas, leading to reduced weight gain and hepatic steatosis on a high-fat diet (59). Hatori et al. divided mice into the NA group (normal diet, ad libitum feeding), the NT group (normal diet, 16:8 fasting from ZT13 to ZT21), the FA group (high-fat diet, ad libitum feeding), and the FT group (high-fat diet, 16:8 fasting). Findings revealed that 16:8 fasting corrected dysregulated expression of circadian genes including *Per1*, *Per2*, *Cry1*, *BMAL1*, *CLOCK*, and *Rev-erbα* induced by the high-fat diet. Regarding glucose metabolism, 16:8 fasting improved glucose tolerance by suppressing *CREB* activity and increasing *Cry* expression, thereby reducing hepatic gluconeogenesis genes such as *Pcx* and *G6pc*. Additionally, TRF diminished lipid synthesis genes like *Fasn* through elevated Rev-erbα expression, consequently reducing fat accumulation (60).

The structure and function of the gut microbiota also exhibit circadian rhythms. Disruption of the host’s circadian rhythm can affect bacterial dysbiosis, triggering obesity and related metabolic disorders. Research by Thaiss et al. indicates that the gut microbiota of both mice and humans undergoes circadian oscillations influenced by feeding rhythms. For instance, the relative abundance of certain bacterial groups (such as *Lactobacillus reuteri* and *Dehalobacterium spp*) exhibits distinct circadian rhythmic variations. Transferring microbiota from mice subjected to jet lag via faecal transplantation to germ-free mice resulted in the development of glucose intolerance and obesity phenotypes in the germ-free recipients. This demonstrates that circadian rhythm disruption induced by jet lag leads to gut microbiota dysbiosis, which in turn promotes glucose intolerance and obesity development (61). IF can remodel the circadian rhythms of the gut microbiota. Zarrinpar et al. randomly assigned 12-week-old male C57BL/6 mice into three groups: the NA group (normal diet, ad libitum feeding), the FA group (high-fat diet, ad libitum feeding), and the FT group (high-fat diet, 16:8 fasting). Following an 8-week intervention, the gut microbiota circadian rhythm in the FA group exhibited abnormal alterations (both *Bacteroidetes* and *Firmicutes* phyla displayed circadian ratios of 1:1), whereas the FT group exhibited a circadian rhythm similar to the normal diet group, with *Bacteroidetes* and *Firmicutes* phyla both exhibiting circadian ratios of 3:1. Concurrently, despite consuming a high-fat diet, the FT group exhibited glucose tolerance comparable to the NA group, indicating a protective effect of TRF on glucose metabolism (62). Similar findings emerged from Ye et al., where 8-week-old male mice were randomly assigned to NA (normal diet ad libitum), FA (high-fat diet ad libitum), and FT (high-fat diet with 8-h time-restricted feeding) groups. After an 8-week intervention, FT mice exhibited significantly less weight gain than FA mice and were comparable to NA mice. Notably, the gut microbiota of FT mice showed time-specific circadian rhythmicity, with Bacteroidetes significantly increased and Firmicutes significantly decreased at ZT20 relative to FA mice, while these differences were not observed at other time points. Concurrently, FT mice exhibited restored circadian rhythmicity of hepatic SIRT1 and PPARα protein expression compared to the disrupted patterns in FA mice, resembling the NA group. However, hepatic Per1 mRNA expression patterns in FT mice remained distinct from NA mice, suggesting that TRF partially, but not completely, restores hepatic circadian gene expression (63). These findings indicate that TRF can directly regulate circadian clock gene expression in peripheral tissues and indirectly influence host circadian rhythms by inducing periodic activity in the gut microbiota (Figure 4).

**Figure 4:**
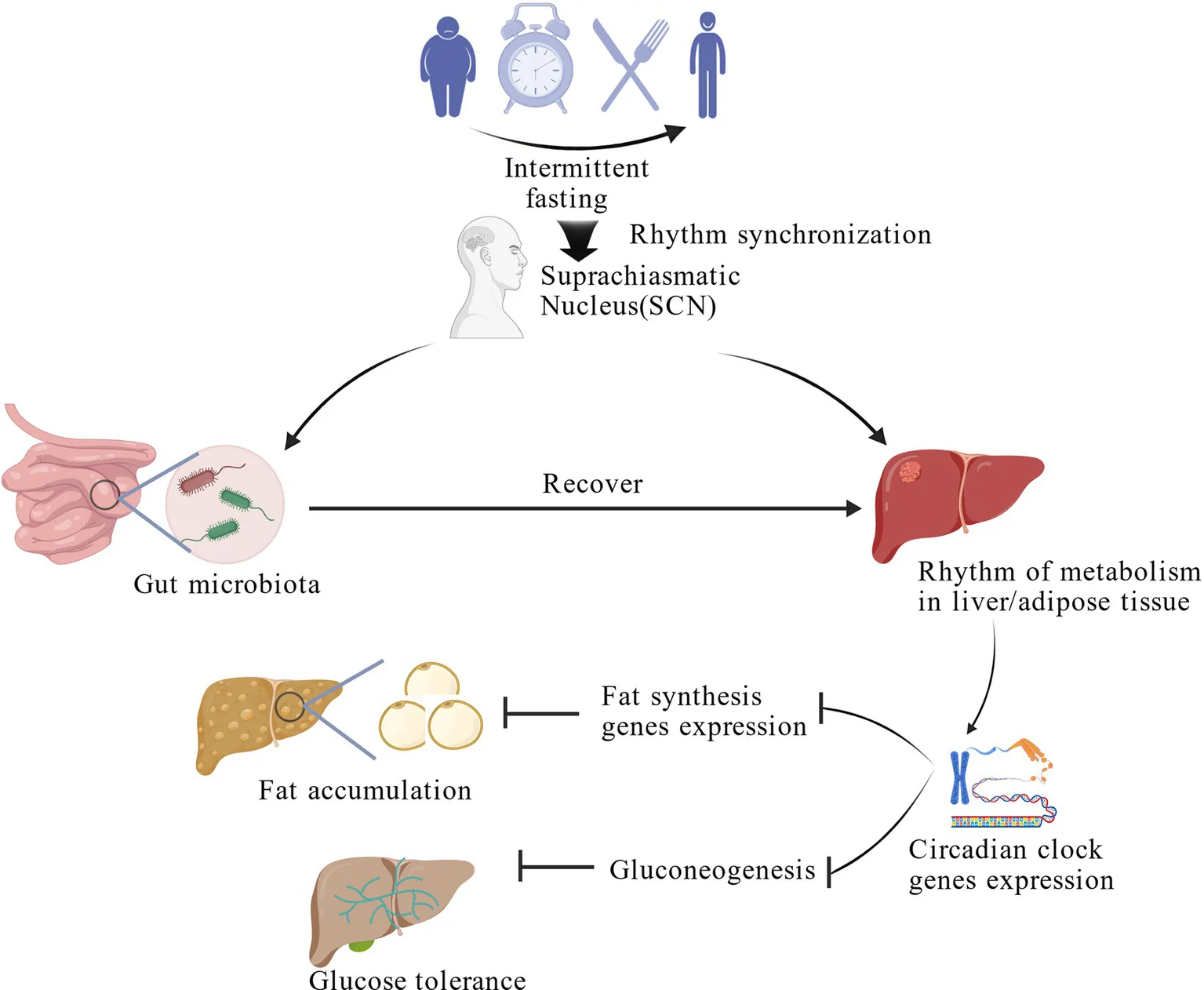
IF regulates circadian gene expression, modulates gut microbiota circadian rhythms, and improves glucose and lipid metabolism. IF restores circadian rhythm synchronization to improve glucose and lipid metabolism. IF acts on the suprachiasmatic nucleus (SCN) and reshapes gut microbiota circadian rhythms, thereby recovering the rhythmic expression of metabolism-related genes in the liver and adipose tissue. This reduces fat synthesis genes (*Fasn*) to decrease fat accumulation, and suppresses gluconeogenesis genes (*Pcx*, *G6pc*) to enhance glucose tolerance. By coordinating central and peripheral circadian oscillators, IF corrects high-fat diet-induced circadian disruption and alleviates obesity-related metabolic disorders.

Despite these compelling preclinical observations, several critical limitations must be acknowledged. First, nearly all mechanistic evidence for circadian resynchronization by IF derives from rodent models; human data demonstrating IF-induced clock gene reprogramming and its causal link to metabolic improvements remain sparse (59). Second, it is unclear whether circadian resynchronization is a primary driver of IF’s metabolic benefits or a secondary consequence of reduced caloric intake and weight loss, as no studies have directly compared IF with CER for circadian outcomes (60). Third, the relative contributions of central (SCN) versus peripheral (liver, adipose, gut) clocks to IF-mediated metabolic improvements have not been systematically dissected (59). Addressing these gaps is essential to determine whether circadian resynchronization represents a mechanism that is selectively engaged by IF and whether it holds translational relevance to human metabolic health.

### 6.3 Gut microbiota-adipose tissue crosstalk: SCFA-induced WAT browning, BAT thermogenesis, and inhibition of intestinal lipid absorption

Adipose tissue thermogenesis involves the production of heat through uncoupling protein 1 (UCP1)-mediated mitochondrial proton leak in brown adipose tissue (BAT) and beige adipocytes, and represents a major determinant of whole-body energy expenditure. Enhancing BAT activity and promoting the browning of WAT have therefore emerged as promising therapeutic strategies for obesity and metabolic disorders. In the context of IF, two conceptually distinct but potentially complementary thermogenic mechanisms have been identified: (1) fasting-induced WAT browning mediated by gut microbiota-derived SCFAs, and (2) feeding-induced (postprandial) BAT thermogenesis, which may be driven by enhanced leptin sensitivity and central neuroendocrine pathways (64).

SCFA-induced WAT browning. Preclinical studies have demonstrated that IF promotes WAT browning through gut microbiota-dependent mechanisms. In mouse models, ADF increased the *Firmicutes/Bacteroidetes* ratio and upregulated the microbial metabolic pathway of pyruvate fermentation to acetate and lactate; these SCFAs increased MCT1 expression, which in turn induced browning of inguinal WAT, as evidenced by upregulation of UCP1, PGC-1α, and PRDM16 (64). Similar browning effects have been observed with *Akkermansia muciniphila* colonization in germ-free mice (65). These findings suggest that IF-mediated shifts in gut microbial composition and SCFA production can promote the conversion of white to beige adipocytes, thereby enhancing energy expenditure. However, it remains unclear whether this mechanism operates in humans, as direct evidence linking IF-induced microbiota changes to WAT browning in human adipose tissue is currently lacking.

Beyond WAT browning, emerging evidence indicates that IF may enhance BAT thermogenesis, particularly during the postprandial (refeeding) period. A recent study in obese mice found that a 16:8 IF regimen increased postprandial oxygen consumption (VO₂), heat production, and BAT temperature (66). These effects were abolished by BAT denervation or UCP1 deletion, confirming their dependence on intact BAT innervation and UCP1-mediated thermogenesis. Notably, serum leptin levels were elevated in IF-treated mice, and the metabolic benefits of 16:8 IF were largely absent in leptin-deficient ob/ob mice, suggesting that enhanced leptin signaling is critical for the thermogenic response. Furthermore, leptin sensitivity was increased in IF-exposed mice, implicating improved central leptin action as a key driver of postprandial thermogenesis.

Leptin as a Central Mediator of Refeeding Thermogenesis. The role of leptin in linking refeeding to thermogenesis is further supported by studies examining postprandial temperature regulation. In 48-h-fasted lean rats, refeeding increased plasma leptin concentrations approximately 8-fold. This surge in leptin stimulates the hypothalamus-adrenal medulla-BAT axis: increased adrenal medullary catecholamine secretion activates β-adrenergic signaling in BAT, which requires fatty acids derived from adipose tissue lipolysis to drive thermogenesis. In diet-induced obese rats, which remain relatively hyperleptinemic even during fasting, this feeding-induced thermogenic response is blunted. Importantly, bolus feeding, which simulates the meal pattern of IF, increased body temperature and reduced weight gain compared with continuous isocaloric feeding; this effect was abrogated by β-adrenergic blockade. Collectively, these findings raise the possibility that IF, by creating cycles of fasting and refeeding, may enhance leptin sensitivity and thereby potentiate postprandial BAT thermogenesis, contributing to improved energy balance beyond the energy deficit incurred during fasting alone (67).

Inhibition of Intestinal Lipid Absorption via the Gut Microbiota-PI3K/AKT Pathway. In addition to its thermogenic effects, IF modulates lipid metabolism through reduced intestinal lipid absorption. The PI3K/AKT signaling pathway regulates intestinal lipid absorption via AKT-mediated phosphorylation and activation of mTORC1, which in turn modulates SREBP1 expression and promotes lipid absorption-associated genes (65). IF suppresses this pathway by altering gut microbiota composition, particularly through increasing the abundance of *Lactobacillus* and *Akkermansia muciniphila*, thereby reducing intestinal lipid uptake (68). This mechanism operates in parallel with WAT browning to limit lipid availability and improve metabolic outcomes (Figure 5).

**Figure 5:**
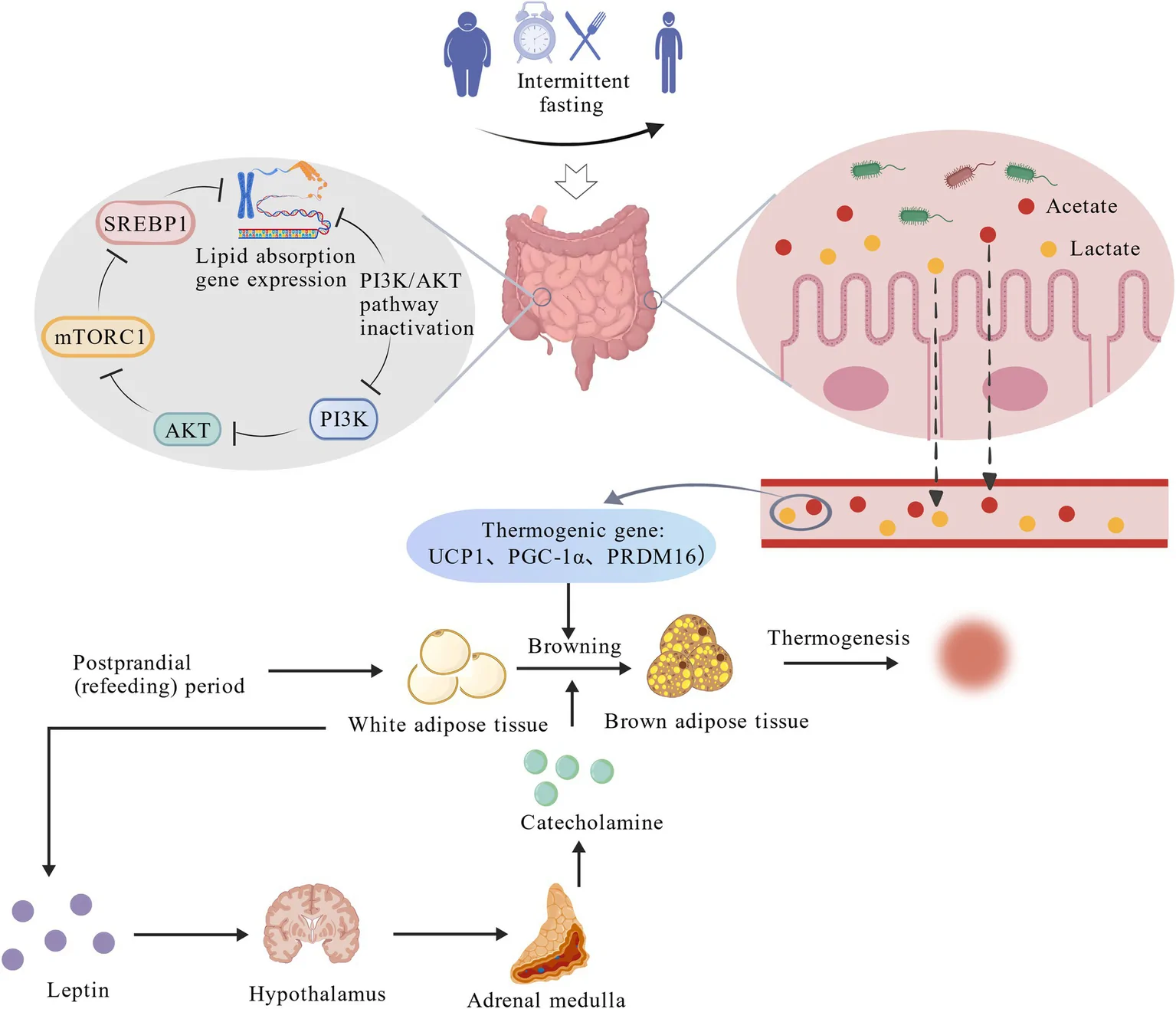
Gut microbiota-dependent mechanisms by which IF promotes WAT browning and inhibits intestinal lipid absorption. IF increases SCFA-producing gut microbes, leading to two parallel effects. SCFAs (acetate, lactate) upregulate MCT1 and activate thermogenic genes (*UCP1, PGC-1α, PRDM16*), inducing WAT browning and enhancing energy expenditure. Concurrently, IF suppresses the PI3K/AKT/mTORC1/SREBP1 pathway, reducing intestinal lipid.absorption. These dual mechanisms collectively improve metabolic homeostasis.

The thermogenic pathways described above, namely SCFA-induced WAT browning (predominantly a fasting/adaptation response) and leptin-mediated BAT thermogenesis (a refeeding/postprandial response), may operate synergistically across the IF cycle to increase overall energy expenditure, while the PI3K/AKT pathway provides an additional mechanism limiting lipid absorption. However, several critical questions remain. First, the relative contribution of WAT browning versus BAT thermogenesis to IF-induced increases in energy expenditure has not been directly compared. Second, while the SCFA-browning and PI3K/AKT pathways have been established in rodent models, their translational relevance to humans is uncertain; no human studies have directly demonstrated IF-induced WAT browning or its dependence on gut microbiota composition. Third, the leptin-BAT thermogenesis pathway has been primarily characterized in acute refeeding paradigms; whether chronic IF regimens sustainably enhance leptin sensitivity and postprandial thermogenesis in humans requires investigation. Addressing these gaps is essential to determine whether the thermogenic effects of IF represent a mechanism that is distinct from, and additive to, the energy deficit imposed by reduced caloric intake.

### 6.4 Cellular autophagy enhancement via the SIRT3-AMPK-mTOR pathway

Autophagy is a cellular degradation process that maintains metabolic homeostasis by removing damaged organelles and recycling intracellular components (69). This process is initiated when cells encounter stimuli such as starvation or oxidative stress. First, double-membrane structures called autophagosomes form within the cell, recognising and engulfing damaged organelles, misfolded proteins, and other substances. These autophagosomes then fuse with lysosomes, where the lysosomal hydrolases degrade the enclosed material. The degradation products can subsequently be reused by the cell (70, 71). During IF, restricted nutrient supply reduces intracellular nutrient and energy levels, elevating the AMP/ATP ratio. This activates the AMP-dependent protein kinase (AMPK) signalling pathway, which directly phosphorylates and activates the autophagy-related protein *ULK1*. AMPK directly phosphorylates and activates ULK1 to initiate autophagosome formation, while simultaneously inhibiting mTORC1 through TSC1/TSC2, thereby relieving mTORC1-mediated suppression of ULK1 (72). This coordinated activation promotes autophagy and enhances cellular utilization of glucose and lipids.

Preclinical studies have demonstrated that IF activates autophagy through the SIRT3-AMPK-mTOR axis. In a rat model, ADF increased autophagosome numbers in skeletal muscle and upregulated AMPKα, phosphorylated AMPK, and ULK1 expression, accompanied by elevated LC3-II/LC3-I ratio and reduced *p62* levels, indicating enhanced autophagic flux (73). In high-fat diet-induced obese mice, ADF upregulated SIRT3 and activated AMPK, and inhibition of SIRT3 attenuated the insulin-sensitizing effects of ADF, confirming that SIRT3 acts upstream of AMPK in this pathway (74). Additionally, ADF has been shown to reduce mTOR phosphorylation in the liver, increase the LC3-II/LC3-I ratio, and decrease p62 expression, hallmarks of autophagy activation, alongside improvements in glucose tolerance and serum triglyceride levels (75). These findings collectively indicate that IF enhances autophagy through SIRT3 upregulation, AMPK activation, and mTORC1 inhibition, thereby facilitating the clearance of damaged cellular components and improving glucose and lipid utilization (Figure 6).

**Figure 6:**
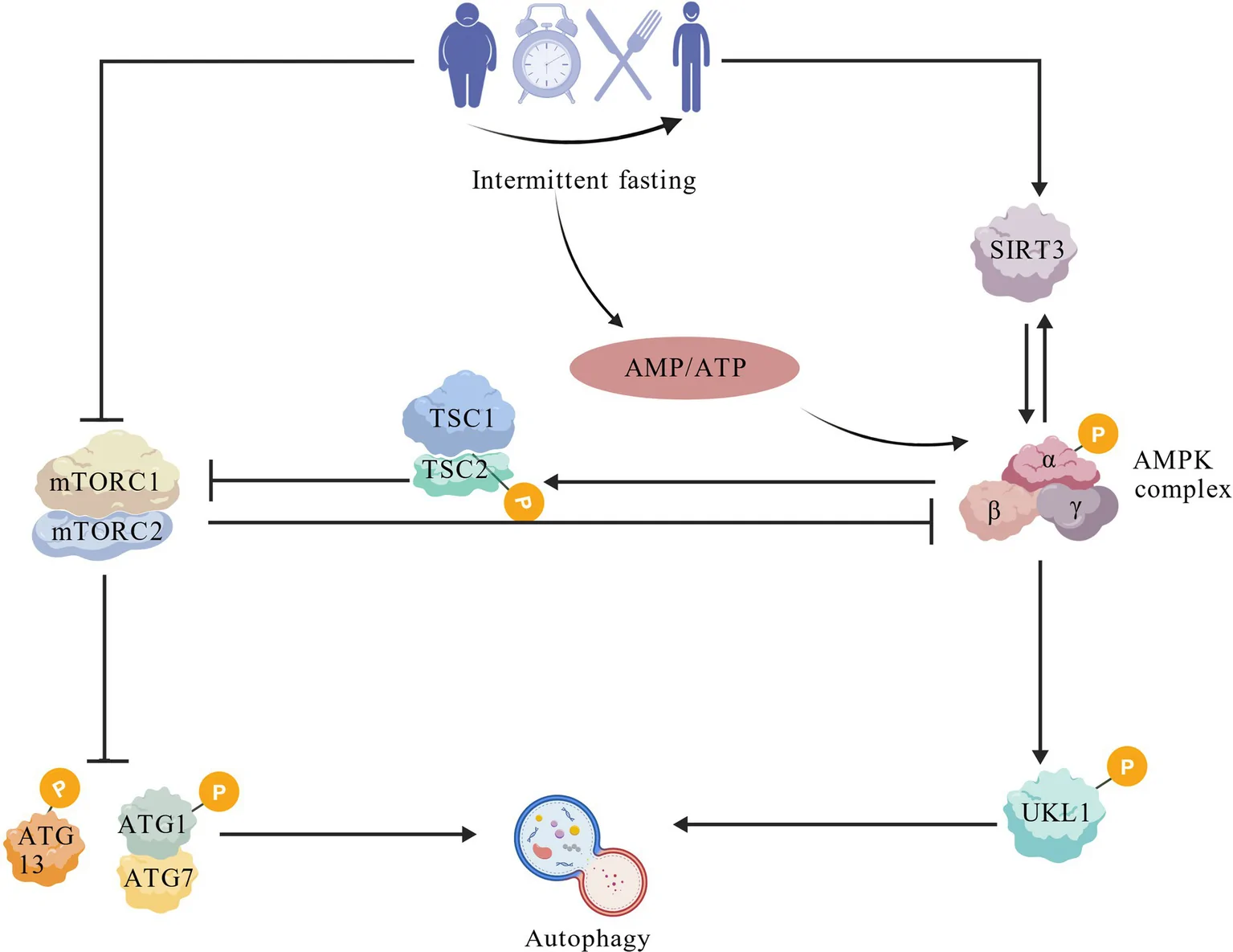
IF induces cellular autophagy via the SIRT3-AMPK-mTOR pathway. IF activates autophagy via the SIRT3-AMPK-mTOR signaling pathway to improve glucose and lipid metabolism. IF upregulates SIRT3 expression and increases the AMP/ATP ratio, which activates the AMPK complex. Activated AMPK phosphorylates ULK1 to initiate autophagosome formation (involving ATG13, ATG7), while concomitantly inhibiting mTORC1 through TSC1/TSC2, thereby relieving mTORC1-mediated suppression of ULK1. The coordinated activation of AMPK and inhibition of mTORC1 synergistically induce autophagy, enhancing cellular clearance of damaged organelles and promoting efficient utilization of glucose and lipids, ultimately improving metabolic homeostasis.

However, several critical limitations must be acknowledged (72, 74). First, all mechanistic evidence for IF-induced autophagy comes from preclinical rodent models; whether IF activates autophagy in human tissues and whether such activation contributes to metabolic improvements in humans remain unproven. Second, it is unclear whether the autophagy-enhancing effects of IF are driven by fasting-specific signals or are simply a consequence of reduced caloric intake, as no studies have directly compared IF with CER for autophagy outcomes. Third, while SIRT3 has been identified as an upstream regulator of AMPK in rodents, the translational relevance of this pathway to humans requires investigation. Addressing these gaps is essential to determine whether autophagy activation is specifically engaged by IF and contributes to its metabolic benefits independently of caloric restriction.

## 7 Conclusion

IF may offer metabolic benefits that extend beyond weight loss, potentially through chronobiological regulation of glucose and lipid homeostasis, although direct comparisons with caloric restriction have yielded mixed findings. It has been shown that IF induces an energy deficit by establishing a time window for food intake, thereby effectively reducing body weight and fat mass. This further improves insulin sensitivity and lipid metabolism parameters, offering a viable nutritional intervention strategy for the prevention and management of metabolic diseases. At the mechanistic level, this paper analyses IF’s pathways for regulating metabolic homeostasis from multiple dimensions, primarily including: reshaping gut microbiota composition to promote short-chain fatty acid production, thereby enhancing intestinal mucosal barrier function; coordinating central and peripheral circadian rhythms to restore synchrony in metabolic pathway diurnal oscillations; inducing browning of WAT to elevate energy expenditure; and enhancing autophagy flux via the SIRT3-AMPK-mTOR signalling axis to improve mitochondrial function and promote metabolic waste clearance. Notably, many of these mechanistic insights derive primarily from preclinical studies, and their translation to humans requires rigorous validation. It should be noted that IF may be associated with adverse reactions such as dizziness, hypoglycaemia, and hair follicle regeneration disorders during implementation. Therefore, a comprehensive assessment of individual metabolic characteristics and tolerance is essential in clinical translation to ensure the safe and effective implementation of personalised patterns. Future research should prioritize large-scale randomized trials to rigorously test these chronobiological mechanisms and identify biomarkers that predict individual responses to IF.
